# InGaN/Dilute-As GaNAs Interface Quantum Well for Red Emitters

**DOI:** 10.1038/srep19271

**Published:** 2016-01-13

**Authors:** Chee-Keong Tan, Damir Borovac, Wei Sun, Nelson Tansu

**Affiliations:** 1 Center for Photonics and Nanoelectronics, Department of Electrical and Computer Engineering, Lehigh University, Bethlehem, PA 18015, USA

## Abstract

The design of InGaN/dilute-As GaNAs interface quantum well (QW) leads to significant redshift in the transition wavelength with improvement in electron-hole wave function overlap and spontaneous emission rate as compared to that of the conventional In_0.2_Ga_0.8_N QW. By using self-consistent six-band ***k·p*** band formalism, the nitride active region consisting of 30 Å In_0.2_Ga_0.8_N and 10 Å GaN_0.95_As_0.05_ interface QW leads to 623.52 nm emission wavelength in the red spectral regime. The utilization of 30 Å In_0.2_Ga_0.8_N/10 Å GaN_0.95_As_0.05_ interface QW also leads to 8.5 times enhancement of spontaneous emission rate attributed by the improvement in electron-hole wavefunction overlap, as compared to that of conventional 30 Å In_0.35_Ga_0.65_N QW for red spectral regime. In addition, the transition wavelength of the interface QW is relatively unaffected by the thickness of the dilute-As GaNAs interface layer (beyond 10 Å). The analysis indicates the potential of using interface QW concept in nitride-based light-emitting diodes for long wavelength emission.

In recent years, III-Nitride semiconductor alloys are regarded as an important semiconductor class in solid state lighting technology. The key advances in the development of III-Nitride-based light emitting diodes (LEDs) has since led to practical implementation in various solid-state lighting applications^1^,^2^,^3^,^4^,^5^,^6^. Owing to the capability of InGaN to emit light in the entire visible spectral regime from blue to red, the realization of monolithic integrated red-green-blue (RGB) GaN-based LEDs will be important towards achieving smart and ultra-efficient solid-state lighting technology^4^.

Despite the success in developing high quality blue and green InGaN LEDs, extending the nitride-based QW emission wavelength towards red spectral regime is fundamentally challenging for two primary reasons. Higher In-content in the InGaN QW is always required in order to achieve the emission wavelength in red spectral regime, but phase separation of the InGaN material occurs simultaneously when the In-content becomes higher^7^,^8^. This leads to crystal degradation in the QW which is detrimental to the optical properties of the InGaN QW LED. Moreover, due to the polarization fields in the InGaN QW, the electron and hole wavefunctions are always spatially separated in the QW [illustrated in Fig. 1(a)] which leads to reduction in the electron-hole wavefunction overlap. The detrimental effect from the charge separation issue in the InGaN QW is increasingly worsening as In-content increases in the QW.

Several approaches have been made to address the charge separation issue in blue and green QW LEDs^9^,^10^,^11^,^12^,^13^,^14^,^15^,^16^,^17^,^18^,^19^, including nonpolar/semipolar InGaN QW^9^, staggered InGaN QW^10^,^11^,^12^ and InGaN QW with AlGaN delta-layer^13^,^14^. However, the approaches are not entirely applicable to addressing the issues in red QW LEDs since high In-content incorporation needs to be taken into consideration. In the case of nonpolar/semipolar InGaN QW, the compensation of quantum confined stark effect in the quantum well leads to blue-shift of emission wavelength if considering a comparison to the c-plane InGaN QW with same In-content^20^. This implies the requirement of higher In-content in nonpolar/semipolar InGaN QW to extend the emission into longer wavelength regime, which results in additional difficulty for the growth of high quality crystal. On the other hand, in the case of staggered InGaN QWs, the electron-hole wavefunction overlap decreases as the emission wavelength extends into longer wavelength such as red spectral regime^11^.

The efforts devoted on extending the nitride-based QW LED emission wavelength towards red spectral regime are still significantly lacking, albeit the research progress is picking up momentum lately. To date a number of approaches have been proposed to address the issues in red emitting GaN-based QW LEDs which include the InGaN metamorphic buffer layer or InGaN substrate for InGaN QW^18^,^19^, the InGaN-delta-InN QW^21^, InGaN with AlGaN interlayer QW^22^, Eu-doped GaN QW instead of InGaN QW^23^,^24^,^25^, lattice-relaxed InGaN multiple QW structure^26^ and semipolar InGaN QW^27^. Note that most approaches in addressing red nitride QW LED are fairly similar to the approaches in addressing the blue and green nitride QW LEDs. Nonetheless the In-content is more than 30% in the InGaN QW for red emission, and the reported highest external quantum efficiency of the red GaN-based QW LED is 2.9%^22^ which is still much lower than that of blue and green GaN-based QW LEDs.

If a high efficiency red emitting nitride-based QW LED can be realized, the making of a RGB QW nitride LED is prospectively achievable. This will provide an alternative solution for the related community to generate white light emission via nitride-based QW LED. Additionally, from the standpoint of science innovations and engineering, it is of great importance to overcome the barriers in achieving high efficiency red emitting nitride QW LED for further revolution in nitride-based solid state lighting technology.

In this work, we present a relatively low In-content nitride-based active region with large electron-hole wavefunction overlap by employing InGaN-GaNAs interface QW concept. The insertion of an interface layer of dilute-As GaNAs alloy adjacent to the InGaN QW layer leads to significantly enhanced electron-hole wavefunction overlap. In contrast to the existing approaches in incorporating high In-content (~35–50%) in the active region for red emission, the In-content in the InGaN QW layer for the InGaN-GaNAs interface QW is relatively small (20%). The characteristics of InGaN-GaNAs QW are presented and are compared to those of the conventional InGaN QW. Note that dilute-As GaNAs alloy has recently been suggested as a potential candidate to be used for LED applications and to suppress interband Auger recombination process which could be important to reduce the efficiency droop issue in the InGaN-based LED devices^28^,^29^,^30^.

## Concept

Our analysis and calculations are carried out based on self-consistent 6-band ***k·p*** formalism for wurtzite semiconductors, in which the valence band mixing, carrier screening effects, polarization fields and strain effect are taken into consideration^17^,^31^,^32^. III-Nitride band parameters are obtained from ref. 33 and conduction to valence band offset ratio (∆E_c_:∆E_v_ = 70:30) is set constant for all layers except for dilute-As GaNAs layer. The material parameters of dilute-As GaNAs layer used in our calculations were obtained through our previous First-Principle Density Functional Theory calculations, including the band properties^28^ and the conduction to valence band offset ratio of GaN/dilute-As GaNAs (∆E_c_:∆E_v_ = 5:95)^29^. In our present study, the focus of the structure employed the GaNAs layer with 5% As-content for aiming at the red emitting active region. The use of 5% As-content in the dilute-As GaNAs layer significantly shifts the valence band edge energy upwards, leading to a reduction of the energy band gap.

The utilization of interface quantum well concept originated from the development in GaAs-based and GaSb-based QW systems^34^,^35^,^36^,^37^,^38^. In conventional GaAs-based QW systems which lacks the spontaneous polarization field, a type-I confinement of the electrons and holes in the QW results in close-to-unity overlap of the electron and hole wavefunctions. In order to elongate the transition wavelength towards mid-infrared spectral regime using GaAs-based and GaSb-based material system, interface quantum well was proposed which then lead to the state-of-the-art laser devices in the infrared regime^34^,^35^,^36^,^37^,^38^. The penalty of interface QW in the GaAs-based or GaSb-based material systems is the reduction of electron-hole wavefunction overlap in the active region, due to the separate confinement of electron and hole wave function in the adjacent layers respectively.

**Figure 1:**
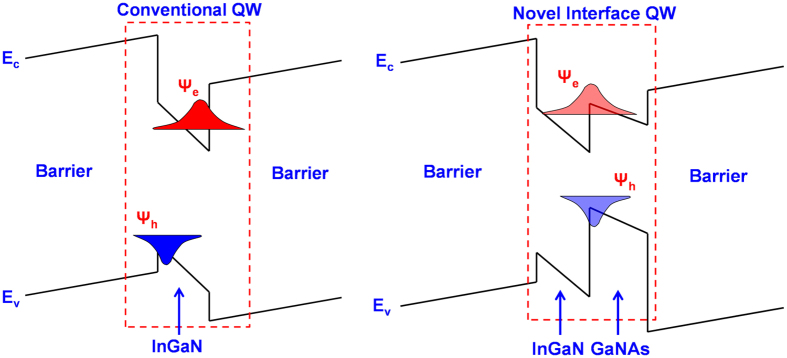
Illustration of (**a**) Conventional nitride-based QW band lineup where the hole and the electron wavefunction are spatially separated in the opposite direction, and (**b**) Novel nitride-based interface QW where the holes and electrons are confined at the interface of two quantum well layers.

On the other hand, as illustrated in Fig. 1(b), the electron and hole wavefunctions are trapped near the hetero-interface in the interface QW structure driven by the large heterojunction discontinuity and the large polarization field mismatch between the InGaN and dilute-As GaNAs layers. As such, the electron wave function and hole wave function will be confined in the first and second layers, respectively, so that the confinement of the wave functions occurs close to the interface of the two material layers. Such carrier localization at the interface would thus dramatically enhance the electron-hole wavefunction overlap in the active region as compared to that of the conventional InGaN QW. Note that a similar concept using type-II “W” QW structure has previously been suggested to suppress the charge separation issue in InGaN QWs^15^,^16^. However, to our knowledge, the interface QW concept is yet to be applied and studied in nitride-based material systems. The type-II “W” QW^15^,^16^ used three layer structures (InGaN/dilute-As GaNAs/InGaN) for achieving improved overlap design, which provided an additional challenge attributed to the need for having three layers grown to form the active region. In this present work, by taking advantage of the large heterojunction discontinuity and the existence of the large polarization field mismatch between the InGaN and dilute-As GaNAs layer, the electron and hole wavefunctions are trapped near the hetero-interface in the interface QW structure.

## Results and Discussion

**Figure 2:**
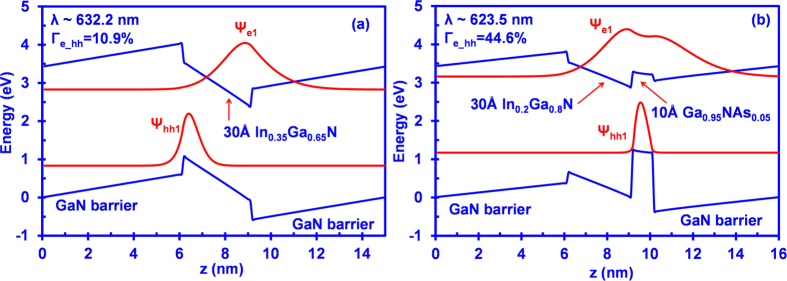
Energy band lineups of (**a**) conventional 30 Å In_0.2_Ga_0.8_N QW, and (**b**) 30 Å In_0.2_Ga_0.8_N/10 Å GaN_0.95_As_0.05_ QW with electron wave function (Ψ_e1_) and hole wave function (Ψ_hh1_).

Figure 2(a,b) show the energy band lineup of conventional 30 Å In_0.35_Ga_0.65_N QW and 30 Å In_0.2_Ga_0.8_N QW coupled with 10 Å GaN_0.95_As_0.05_ interface-layer respectively, along with the plotted wavefunctions of first conduction subband and first valence subband in both QW structures. Both structures are designed for transition wavelength of 620-630 nm for red spectral regime. As can be seen in Fig. 2(a), the existence of an internal electric field leads to the tilted band lineup across the structure, causing the charge separation such that the hole wavefunction is pulled towards the left side while the electron wavefunction is pulled towards the right side. The spatially separated electron and hole wavefunctions accordingly lead to significantly reduced overlap of 10.9% between the wavefunctions for conventional 30 Å In_0.35_Ga_0.65_N QWs.

On the other hand, as shown in Fig. 2(b), by using a lower In-content (20%-In) for the InGaN QW layer and by coupling the InGaN QW layer with 10 Å GaN_0.95_As_0.05_ interface layer in the active region, the large electron-hole wavefunction overlap could be achieved for the QW structure. The large valence band offset between InGaN and GaNAs layers leads to strong hole localizations towards the GaNAs layer, while the electron wavefunction is extended beyond the active region for structure employing GaN as the barrier layers. The wavefunction overlap in the interface QW structure therefore results in the enhanced electron-hole wavefunction overlap of 44.6%. The natural band alignment for dilute-As GaNAs/GaN results in ∆E_c_:∆E_v_ = 5:95^29^. It is important to note that the inclusion of strain effect in the dilute-As GaNAs resulted in the “weak” type-II band alignment in dilute-As GaNAs/GaN interface attributed to its upward shift of the conduction band edge.

**Figure 3:**
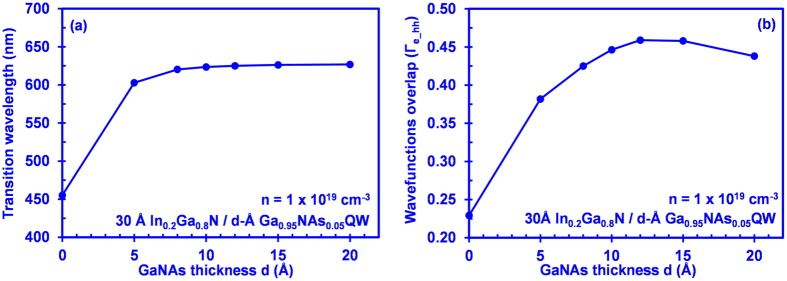
(**a**) Interband transition wavelength and (**b**) electron-hole wave function overlap as a function of GaNAs layer thickness for 30 Å In_0.2_Ga_0.8_N/d-Å GaN_0.95_As_0.05_ QW at carrier density of 1 × 10^19^ cm^−3^.

Figure 3(a) shows the interband transition wavelength as a function of the GaNAs layer thickness (d) for 30 Å In_0.2_Ga_0.8_N/d-Å GaN_0.95_As_0.05_ QW structure at carrier density of 1 × 10^19^ cm^−3^. As shown in Fig. 3(a), the transition wavelength of the QW structure increases rapidly from 455.2 nm to 623.52 nm as the thickness of the GaNAs layer increases. The interband transition wavelength remains relatively unchanged in the ~625 nm spectral regime when the thickness of the GaNAs layer increases from 10 Å to 20 Å, which is attributed to the deep hole localization in the GaNAs layer. Figure 3(b) shows the electron-hole wavefunction overlap as a function of the GaNAs layer thickness for the 30 Å InGaN/d-Å GaNAs QW structure at carrier density of 1 × 10^19^ cm^−3^. In general, the electron-hole wavefunction overlap increases as the GaNAs layer thickness increases due to the shifting of the electron and hole wavefunction towards the interface of the two-layer QW. However, the electron-hole wavefunction overlap of the InGaN-GaNAs QW decreases slightly from 45% to 42.5% when the thickness of the GaNAs layer increases from 15 Å to 20 Å. Note that the 30 Å In_0.2_Ga_0.8_N QW without the GaNAs layer will yield a transition wavelength of 455 nm and wavefunction overlap of 22.9% which is considerably low as compared to that of the 30 Å In_0.2_Ga_0.8_N/d-Å GaN_0.95_As_0.05_ QW structure. As a comparison, even though 30 Å In_0.35_Ga_0.65_N QW yields a transition wavelength of 632 nm, the wavefunction overlap is small (10.9%), which is 4 times smaller than that of 30 Å In_0.2_Ga_0.8_N/d-Å GaN_0.95_As_0.05_ QW structure.

Note that the designed structure of the InGaN QW with the coupling of the dilute-As GaNAs layer allows one to have transition wavelength redshift from blue spectral regime to red spectral regime and significantly enhanced electron-hole wavefunction overlap in the QW as compared to the conventional InGaN QW with same Indium composition and same InGaN QW thickness. In addition, the interband transition wavelength of the interface QW structure is relatively insensitive to the thickness of GaNAs layers beyond 10 Å as can be seen in Fig. 3(a), which is different from the InGaN-delta-InN QW structure^21^ where the resulting interband transition wavelength is highly dependent on the InN QW thickness. Accordingly, the similar redshifted transition wavelength (in this case blue to red spectral regime) independent of the varying interface layer thickness indicates the high potential of using interface QW concept in realizing highly controllable transition wavelength with large electron-hole wavefunction overlap under state-of-the-art growth technology.

**Figure 4:**
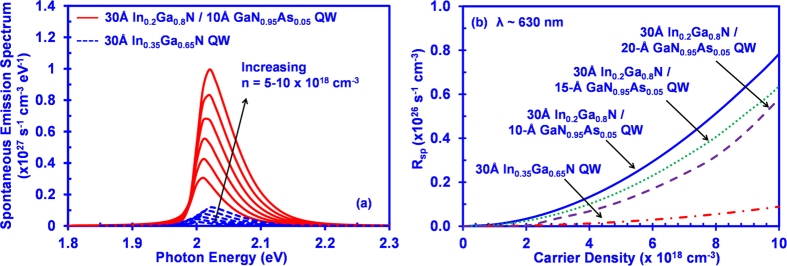
(**a**) Spontaneous emission spectra as a function of photon energy with carrier density from 5 × 10^18^ cm^−3^ to 1 × 10^19^ cm^−3^ for conventional 30 Å In_0.35_Ga_0.65_N QW and 30 Å In_0.2_Ga_0.8_N/10 Å GaN_0.95_As_0.05_ QW and (**b**) Spontaneous emission rate R_sp_ as a function of carrier density for conventional 30 Å In_0.35_Ga_0.65_N QW, 30 Å In_0.2_Ga_0.8_N/10 Å GaN_0.95_As_0.05_ QW, 30 Å In_0.2_Ga_0.8_N/15 Å GaN_0.95_As_0.05_ QW, and 30 Å In_0.2_Ga_0.8_N/20 Å GaN_0.95_As_0.05_ QW at T = 300 K.

Figure 4(a) shows a comparison of spontaneous emission spectra for conventional 30 Å In_0.35_Ga_0.65_N QW (dash line) and 30 Å In_0.2_Ga_0.8_N/10 Å GaN_0.95_As_0.05_ QW (solid line) at carrier densities from 5 × 10^18^ cm^−3^ to 1 × 10^19^ cm^−3^ at T = 300 K. As shown in Fig. 4(a), the spontaneous emission rates for the conventional 30 Å In_0.35_Ga_0.65_N QW is much smaller than that of the 30 Å In_0.2_Ga_0.8_N/10 Å GaN_0.95_As_0.05_ QW. The spontaneous emission rate of 30 Å In_0.2_Ga_0.8_N/10 Å GaN_0.95_As_0.05_ QW is enhanced by 8.5 times as compared to that of conventional 30 Å In_0.35_Ga_0.65_N QW. Specifically, at n = 1 × 10^19^ cm^−3^, the peak spontaneous emission rate of conventional 30 Å In_0.35_Ga_0.65_N QW reaches 1.17 × 10^26^ s^−1^ cm^−3^ eV^-1^ whereas the peak spontaneous emission rate of 30 Å In_0.2_Ga_0.8_N/10 Å GaN_0.95_As_0.05_ QW reaches 9.95 × 10^26^ s^−1^ cm^−3^ eV^-1^.

The improvement in the spontaneous emission recombination rates for the 30 Å In_0.2_Ga_0.8_N/10 Å GaN_0.95_As_0.05_ interface QW could be attributed to the improved hole wavefunction confinement in the GaNAs layer, resulting in the improvement in the electron-hole wavefunction overlap. As a side note, our findings indicate blueshift of transition wavelength for both structures when the carrier density increases. Nevertheless the wavelength blueshift of 30 Å In_0.2_Ga_0.8_N/10 Å GaN_0.95_As_0.05_ QW is considerably smaller than that of the conventional 30 Å In_0.35_Ga_0.65_N QW when the carrier density increases from 5 × 10^18^ cm^−3^ to 1 × 10^19^ cm^−3^. The improvement in the wavelength blueshift for 30 Å In_0.2_Ga_0.8_N/10 Å GaN_0.95_As_0.05_ QW could be attributed to the reduction of the quantum confined stark effect in the active region.

Figure 4(b) shows the spontaneous emission radiative recombination rate per unit volume (R_sp_) for conventional 30 Å In_0.35_Ga_0.65_N QW (dash-dot), 30 Å In_0.2_Ga_0.8_N/10 Å GaN_0.95_As_0.05_ QW (solid), 30 Å In_0.2_Ga_0.8_N/15 Å GaN_0.95_As_0.05_ QW (dot) and 30 Å In_0.2_Ga_0.8_N/20 Å GaN_0.95_As_0.05_ QW (dash) as a function of carrier density up to 10 × 10^18^ cm^−3^. As shown in Fig. 4(b), the R_sp_ of 30 Å In_0.2_Ga_0.8_N/d- Å GaN_0.95_As_0.05_ QW is enhanced at each carrier density as compared to that of conventional 30 Å In_0.35_Ga_0.65_N. Specifically, the enhancement of R_sp_ for the 30 Å In_0.2_Ga_0.8_N/10 Å GaN_0.95_As_0.05_ QW ranges from 8.8–11.3 times.

Note that dilute-As GaNAs alloy has been reported for material growth using metal organic chemical vapor deposition (MOCVD)^39^,^40^ and molecular beam epitaxy (MBE)^41^. In the perspective of MOCVD growth, the growth of dilute-As GaNAs thin film was operated at 700–750 °C^40^ which is comparable to the growth temperature of InGaN QW. Recent experimental studies by MBE have also demonstrated the capability to grow full As composition GaNAs alloy^41^. However, the implementation of GaNAs material in the active region for emitters is yet to be realized up to present. The field of dilute-As GaNAs is still in the early stage due to the lack of clear motivation on the importance of this material system for development of visible emitters. The identification of the dilute-As GaNAs alloy as a promising active material for red emitters with large spontaneous emission rate, as well as our recent work on identifying the non-resonant Auger process in this alloy, will provide a clear and strong motivation on the importance of the experimental pursuit of this material system. Future experimental studies are required to evaluate the performance and advantages presented in the InGaN/dilute-As GaNAs interface QW LEDs.

It is important to also mention that the main idea of this work is to illustrate the advantage of using an interface layer to extend the emission wavelength, while enhancing the matrix element and radiative recombination rates in the QW as compared to the conventional InGaN QW. By controlling the As-content in the dilute-As GaNAs interface layer, it could be expected that the proposed structure of InGaN/GaNAs QW in this study has a high potential for control of the emission wavelength in green and red spectral regime with enhanced matrix element and radiative recombination rates in the QW. Further study will be carried out to investigate the impact of the proposed interface QW on green and yellow spectral regime.

## Conclusion

In summary, the design of interface QW consisting of InGaN layer and dilute-As GaNAs layer leads to the emission wavelength in red spectral regime, along with enhanced electron-hole wavefunction overlap and spontaneous emission rate. Specifically, the design of the 30 Å In_0.2_Ga_0.8_N with 10 Å GaN_0.95_As_0.05_ interface QW results in significant emission wavelength redshift from 455.2 nm to 623.52 nm. Moreover, the spontaneous emission rate of 30 Å In_0.2_Ga_0.8_N/10 Å GaN_0.95_As_0.05_ interface QW is greatly enhanced by 8.5 times as compared to that of the conventional 30 Å In_0.35_Ga_0.65_N QW for red emission. In addition, the emission wavelength of the InGaN/GaNAs interface QW is relatively insensitive to the variation of dilute-As GaNAs interface layer thickness beyond 10 Å, indicating the potential of easing the difficulty for precise control of layer thickness during the epitaxial growth. Therefore the design of interface QW such as InGaN/dilute-As GaNAs QW has the high potential to be used for achieving high-efficiency nitride-based LEDs in red spectral regime.

## Additional Information

**How to cite this article**: Tan, C.-K. *et al.* InGaN/Dilute-As GaNAs Interface Quantum Well for Red Emitters. *Sci. Rep.* **6**, 19271; doi: 10.1038/srep19271 (2016).

## References

[b1] Krames M. *et al.* Status and future of high-power light-emitting diodes for solid-state lighting, J. Disp. Technol., 3, 160–175 (2007).

[b2] Crawford M. H. LEDs for solid-state lighting: performance challenges and recent advances, IEEE. J. Sel. Top. Quantum Electron., 15, 1028–1040 (2009).

[b3] Tansu N. *et al.* III-Nitride Photonics, IEEE Photonics Journal, 2, 241–248 (2010).

[b4] Tsao J. Y. *et al.* Toward smart and ultra-efficient solid-state lighting, Adv. Opt. Mat., 2, 809–836 (2014).

[b5] Tan C. K. & Tansu N. Nanostructured Lasers: Electrons and Holes Get Closer, Nature Nanotechnology, 10, 107–109 (2015). 10.1038/nnano.2014.333 25599192

[b6] Pust P., Schmidt P. J. & Schnick W. A revolution in lighting, Nature Mater., 14, 454–458 (2015). 25899898 10.1038/nmat4270

[b7] Belyaev K. G. *et al.* Phase separation in InxGa1-xN (0.10 < x < 0.40), Phys. Status Solidi C, 10, 527–531 (2013).

[b8] McCluskey M. D. *et al.* Phase separation in InGaN multiple quantum wells, Appl. Phys. Lett., 72, 1730–1732 (1998).

[b9] Feezell D. F., Speck J. S., DenBaars S. P. & Nakamura S. Semipolar (20^-^2^-^1) InGaN/GaN light emitting diodes for high-efficiency solid-state lighting, J. Disp. Tech., 9, 190–198 (2013).

[b10] Arif R. A., Ee Y. K. & Tansu N., Polarization engineering via staggered InGaN quantum wells for radiative efficiency enhancement of light emitting diodes, Appl. Phys. Lett., 91, 091110 (2007).

[b11] Arif R. A., Zhao H. P., Ee Y. K. & Tansu N. Spontaneous emission and characteristics of staggered InGaN quantum-well light-emitting diodes, IEEE J. Quantum Electron., 44, 573–580 (2008).

[b12] Zhao H. P. *et al.* Approaches for high internal quantum efficiency green InGaN light-emitting diodes with large overlap quantum wells, Optics. Express, 19, A991–A1007 (2011). 21747571 10.1364/OE.19.00A991

[b13] Park J. & Kawakami Y. Photoluminescence property of InGaN single quantum well with embedded AlGaN delta layer, Appl. Phys. Lett., 88, 202107 (2006).

[b14] Park S. H., Park J. & Yoon E. Optical gain in InGaN/GaN quantum well structures with embedded AlGaN delta layer, Appl. Phys. Lett., 90,. 023508 (2007).

[b15] Arif R. A., Zhao H. P. & Tansu N. Type-II InGaN-GaNAs quantum wells for lasers applications, Appl. Phys. Lett., 92, 011104 (2008).

[b16] Zhao H. P., Arif R. A. & Tansu N. Self-consistent gain analysis of type-II ‘W’ InGaN-GaNAs quantum well lasers, J. Appl. Phys., 104, 043104 (2008).

[b17] Zhao H. P., Arif R. A., Ee Y. K. & Tansu N. Self-consistent analysis of strain-compensated InGaN-AlGaN quantum wells for lasers and light emitting diodes, IEEE J. Quantum Electron., 45, 66–78 (2009).

[b18] Zhang J. & Tansu N. Improvement in spontaneous emission rates for InGaN quantum wells on ternary InGaN substrate for light-emitting diodes, J. Appl. Phys., 110, 113110 (2011).

[b19] Daubler J. *et al.* Long wavelength emitting GaInN quantum wells on metamorphic GaInN buffer layers with enlarged in-plane lattice parameter, Appl. Phys. Lett., 105, 111111 (2014).

[b20] Avramescu A. *et al.* True green laser diodes at 524 nm with 50 mW continuous wave output power on c-plane GaN, Appl. Phys. Express, 3, 061003 (2010).

[b21] Zhao H. P., Liu G. Y. & Tansu N. Analysis of InGaN-delta-InN quantum wells for light-emitting diodes, Appl. Phys. Lett., 97, 131114 (2010).

[b22] Hwang J., Hashimoto R., Saito S. & Nunoue S. Development of InGaN-based red LED grown on (0001) polar surface, Appl. Phys. Express, 7, 071003 (2014).

[b23] Heikenfeld J., Garter M., Lee D. S., Birkhahn R. & Steckl A. J. Red light emission by photoluminescence and electroluminescence from Eu-doped GaN, Appl. Phys. Lett., 75, 1189–1191 (1999).

[b24] Nishikawa A., Furukawa N., Kawasaki T., Terai Y. & Fujiwara Y. Room-temperature red emission from light-emitting diodes with Eu-doped GaN grown by organometallic vapor phase epitaxy, Optical Materials 33, 1071–1074 (2011).

[b25] Nishikawa A., Kawasaki T., Furukawa N., Terai Y. & Fujiwara Y. Room-temperature red emission from a p-type/Europium-doped/n-type gallium nitride light-emitting diode under current injection, Appl. Phys. Express, 2, 071004 (2009).

[b26] Ohkawa K., Watanabe T., Sakamoto M., Hirako A. & Deura M. 740-nm emission from InGaN-based LEDs on c-plane sapphire substrates by MOVPE, J. Crys. Growth, 343, 13–16 (2012).

[b27] Kawaguchi Y. *et al.* Semipolar (2021) single-quantum-well red light-emitting diodes with a low forward voltage, Jap. J. Appl. Phys., 52, 08JC08 (2013).

[b28] Tan C. K. *et al.* First-Principle electronic properties of dilute-As GaNAs alloy for visible light emitters, J. Disp. Tech., 9, 272–279 (2013). 10.1038/srep24412 PMC4830964 27076266

[b29] Tan C. K. & Tansu N. First-Principle natural band alignment of GaN/dilute-As GaNAs alloy, AIP Advances, 5, 071129 (2015).

[b30] Tan C. K. & Tansu N. Auger Recombination Rates in Dilute-As GaNAs Semiconductor, AIP Advances, 5, 057135 (2015).

[b31] Chuang S. L. & Chang C. S. k.p method for strained wurtzite semiconductors, Phys. Rev. B, 54, 2491–2504 (1996). 10.1103/physrevb.54.2491 9986096

[b32] Chuang S. L. Optical gain of strained wurtzite GaN quantum-well lasers, IEEE J. Quantum Electron., 32, 1791–1800 (1996).

[b33] Vurgaftman I. & Meyer J. R. Nitride semiconductor Devices, Piprek J., Ed. ch. 2, New York: Wiley, 2007.

[b34] Meyer J. R., Hoffman C. A., Bartoli F. J. & Ram-Mohan L. R. Type-II quantum-well lasers for mid-wavelength infrared, Appl. Phys. Lett., 67, 757–759 (1995).

[b35] Vurgaftman I. *et al.* Mid-IR type-II interband cascade lasers, IEEE J. Selected Topics in Quantum Electronics, 17, 1435–1444 (2011).

[b36] Yang R. Q. *et al.* High power mid-infrared interband cascade lasers based on type-II quantum wells, Appl. Phys. Lett., 71, 2409–2411 (1997).

[b37] Vurgaftman I., Meyer J. R., Tansu N. & Mawst L. J. (In)GaAsN-based type-II “W” quantum-well lasers for emission at λ = 1.55 μm, Appl. Phys. Lett., 83, 2742–2744 (2003).

[b38] Bank S. R., Goddard L. L., Wistey M. A., Yuen H. B. & Harris J. S. On the temperature sensitivity of 1.5 μm GaInNAsSb lasers, IEEE J. Sel. Topics Quantum Electron., 11, 1089–1098 (2005).

[b39] Li X., Kim S., Reuter E. E., Bishop S. G. & Coleman J. J. The incorporation of arsenic in GaN by metalorganic chemical vapor deposition, Appl. Phys. Lett., 72, 1990–1992 (1998).

[b40] Kimura A., Paulson C. A., Tang H. F. & Kuech T. F. Epitaxial GaN1-yAsy layers with high As content grown by metalorganic vapor phase epitaxy and their band gap energy, Appl. Phys. Lett., 84, 1489–1491 (2004).

[b41] Yu K. M. *et al.* Non-equilibrium GaNAs alloys with band gap ranging from 0.8–3.4 eV, Phys. Status Solidi C, 7, 1847–1849 (2010).

